# The Factorization Method for Electrical Impedance Tomography Data from a New Planar Device

**DOI:** 10.1155/2007/83016

**Published:** 2007-06-03

**Authors:** Mustapha Azzouz, Martin Hanke, Chantal Oesterlein, Karl Schilcher

**Affiliations:** ^1^Institut für Physik, Johannes Gutenberg-Universität Mainz, 55099 Mainz, Germany; ^2^Institut für Mathematik, Johannes Gutenberg-Universität Mainz, 55099 Mainz, Germany

## Abstract

We present numerical results for two reconstruction methods for a new planar electrical impedance tomography device. This prototype allows noninvasive medical imaging techniques if only one side of a patient is accessible for electric measurements. The two reconstruction methods have different properties: one is a linearization-type method that allows quantitative reconstructions; the other one, that is, the factorization method, is a qualitative one, and is designed to detect anomalies within the body.

## 1. INTRODUCTION

In electrical impedance tomography (EIT), electric currents are applied to the boundary of an object and the induced surface voltages are measured. These data are used to reconstruct the conductivity distribution in the interior of the object. Important practical applications arise in medical imaging.

Unfortunately, this *inverse conductivity problem* is nonlinear and severely ill-posed. That means that even large conductivity variations in the interior of the object may only lead to tiny changes in the surface data. EIT therefore represents a challenging problem. Most reconstruction procedures that have been proposed include either some iterative or some linearization methods where the ill-posedness is regulated in one way or another (cf., e.g., Holder [1]). It is fair to say that the success in medical applications is rather disappointing, except possibly for two-dimensional situations.

A way out of this dilemma is to reduce the amount of information that is to be extracted from the data by incorporating a priori information. In many applications, for example, in mammography, one may only be interested in finding regions in the interior where the conductivity changes rapidly in comparison to an approximately constant background conductivity. In this way, the ill-posedness is circumvented to a certain extent.

The so-called *factorization method* is a reconstruction scheme that is adapted to this kind of applications. In this note, we present some first numerical results for this particular method using real EIT data. These data were obtained with a new EIT device developed at our institution which allows to take data in two- and three-dimensional configurations.

In contrast to most previous EIT instruments, but similar to prototypes studied, for example, by Mueller [2], and by Cherepenin [3], our device uses a planar sensing head, and is designed for medical applications where measurements can only be taken from one side of the patient. This is an important issue in the context of mammography, or in monitoring patients in intensive care units, to name only two such applications. We refer to Zou and Guo [4] for a review of further electrical impedance imaging devices for breast cancer detection, including the commercial TS 2000 system.

## 2. DESCRIPTION OF THE DEVICE

**Figure 1:**
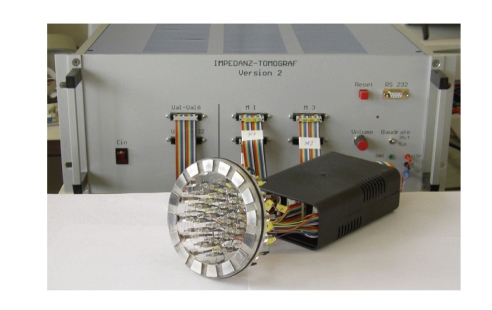
The tomograph.

The Mainz tomograph consists of three parts, a sensing head, an electronic device to apply and measure electric potential and current patterns, and a computer for the image reconstruction (cf. Figure 1).

The sensing head has a diameter of 10cm and consists of 16 large electrodes, arranged on the outer ring of a disk. Through these electrodes the current is injected and both current and voltages can be measured. There is another set of 64 small high-impedance electrodes placed in the interior which can be used to measure additional voltages, however, these measurements have not yet been used to solve the inverse problem.

The data acquisition device consists of five modules. The first module is a microcontroller to facilitate the communication between the measuring device and the external components via an RS232 serial interface. The second module generates preset sinusoidal voltages of frequency 5–50 kHz which can be used to drive 16 (or 32) current injecting electrodes. The amplitudes can be set positive or negative by 32 DAC to 16-bit accuracy and can be modulated program-controlled to any desired amplitude pattern. The resulting current at each injection electrode passes through a precision resistor and a special operational amplifier to facilitate the simultaneous measurement of the current. The voltages on the interior electrode are measured with 16-bit accuracy with the help of the third module. The fourth module serves to read the data and to measure the signal by a peak detector largely in parallel via 8 multiplexers of 16 channels each. The measured value of the peak detector is subsequently digitalized to 16-bit accuracy. In the fifth module, finally, the sign of the modulation is defined.

There are *N* − 1 independent measurements for *N* current injecting electrodes. In most reconstruction algorithms, it is assumed that currents are prescribed and voltages are measured. Because of the much simpler electronic implementation, voltages are applied and currents are measured on the injectors (voltages are also measured in the interior) in our device. It is, however, no problem to convert to current-driven data by linear combination of the various voltage-driven data. For optimal resolution, it is of advantage to apply trigonometric voltage or current patterns of different frequencies (cf., e.g., Newell et al. [5]).

So far we have no clinical data at our disposal. Instead, we have placed the sensing head into the bottom of an appropriate container of large lateral dimensions and filled with a conducting liquid. The level of the liquid in the tank has been kept very low so as to approximate a two-dimensional situation. Various objects have been immersed into the liquid. Measurements have been taken with and without the immersed bodies. The latter serves as reference measurements where necessary. Below we will present numerical tests with real data that have been obtained this way.

## 3. A LINEARIZATION-TYPE RECONSTRUCTION METHOD

For an isotropic conductivity distribution *σ* = *σ*(**x**) the electric potential *u* = *u*(*x*) satisfies the equation

(1) ∇⋅(σ∇u)=0,

where *σ* and *u* are defined in a certain volume Ω. This is a direct consequence of the conservation of the electric current *j* = *σ* ∇ *u*. A driving force for nontrivial potentials *u* is, for example, a current that is injected into Ω through some electrodes attached to a subset Γ ⊂ *∂*Ω. The above differential equation is fundamental to the study of EIT. For constant conductivities *σ* it becomes the homogeneous Laplace equation, whose solutions *u* are the harmonic functions.

In our application where the device is much smaller than the human body to which it is applied, it appears appropriate to model the volume Ω as a three-dimensional half space, and the subset Γ as a circle, say the unit circle. In fact, as our currents do not penetrate deep into the body, it is even appropriate to further simplify the model, and to identify Ω with its boundary *∂*Ω. This results in a two-dimensional model described by the following diffraction problem:

(2) $$\nabla \cdot (\sigma \nabla u)=0\text{in}{\mathbb{R}}^{2}\backslash \Gamma ,$$

where *u* is constraint to satisfy

(3) $$\begin{array}{c} {\left[\nu \cdot j\right]}_{\Gamma }=f,\;\;{\left[u\right]}_{\Gamma }=0,\\ |u(x)|=O(1)\;\text{as}|x|\to \infty , \end{array}$$

4 $$\int_{\Gamma }\;uds=0.$$

The square brackets in (3) refer to the jump of the respective quantity within the brackets when moving from the inside to the outside of Γ; *ν* is the outer normal of Γ. The first condition in (3) therefore asserts that current *f* is injected into the body along Γ, whereas the second condition requires that *u* is continuous across Γ, that is, that the potential on Γ is well-defined. The last condition in (3) eliminates nonphysical solutions, whereas the physical one is unique after fixing the ground potential; this is accomplished via the normalization condition (4).

In the EIT problem known currents are injected and the resulting potentials are measured on Γ. From these data the conductivity *σ* is to be determined. The currents and potentials on the electrodes are related through the measurement operator

(5) $$M:\{\begin{array}{c} {ℒ}_{⋄}^{2}(\Gamma )\to {ℒ}_{⋄}^{2}(\Gamma ),\\ f\mapsto u{|}_{\Gamma }, \end{array}$$

associated with the differential equation (2), (3), and (4). Here,

(6) $${ℒ}_{⋄}^{2}(\Gamma )=\{g\in {ℒ}^{2}(\Gamma ):\int_{\Gamma }gds=0\}$$

denotes the set of all square integrable functions over Γ with zero mean. Note that we have fixed *u*|Γ to belong to ${ℒ}_{⋄}^{2}(\Gamma )$ according to (4).

For a simple example let *σ* = 1 be constant in ℝ^2^, in which case we will further on write *M*_0_ for *M* of (5). Later on, *σ* = 1 will be considered as our reference, or background conductivity. The associated potential *u* = *u*_0_ is a solution of the homogeneous Laplace equation inside (and outside) the unit disk, and, using polar coordinates, can be written in the form

(7) $$u_{0}(r,\theta )=\sum_{k=-\infty }^{\infty }c_{k}r^{\left|k\right|}e^{ik\theta },0\leq r\leq 1,$$

with complex coefficients *c* *_k_*, *k* ∈ ℤ . On Γ we have

(8) $$u_{0}(1,\theta )=\sum_{k=-\infty }^{\infty }c_{k}e^{ik\theta },$$

and, because of the continuity of the potential across Γ, we obtain

(9) $$u_{0}(r,\theta )=\sum_{k=-\infty }^{\infty }c_{k}r^{-|k|}e^{ik\theta },1<r<\infty .$$

In fact, the normalization (4) requires that *c*_0_ = 0, and hence, it follows from (3) that

(10) $$f(\theta )=[\nu \cdot j{]}_{\Gamma }=[\frac{\partial u_{0}(r,\theta )}{\partial r}{]}_{r=1}=-2\sum_{k\neq 0}|k|c_{k}e^{ik\theta }.$$

The eigenfunctions of *M*_0_ are therefore

(11) $$f^{\left(k\right)}(\theta )=e^{ik\theta },k\in \mathbb{Z}\backslash \{0\},$$

which are mapped onto the potentials

(12) $$u_{0}^{\left(k\right)}(r,\theta )=-\frac{1}{2|k|}\{\begin{array}{cc} r^{\left|k\right|}e^{ik\theta }, & 0\leq r\leq 1,\\ r^{-|k|}e^{ik\theta }, & 1<r<\infty . \end{array}$$

The corresponding eigenvalues of *M*_0_ are given by − 1/( 2 | *k* | ), respectively.

We now turn to the solution of our inverse problem associated with the diffraction problem (2), (3), and (4), namely, the identification of *σ* given the measurement operator *M* of (5). We will compare two different algorithms to solve this problem. First, we use a somewhat standard reconstruction method which is based on a certain kind of linearization, and which is close to the method implemented in [2]; second, we apply the factorization method described in the subsequent section. For both methods we will assume that the conductivity fulfills *σ* = 1 near *Γ* and near infinity.

If *σ* is smooth, (2) can also be written in the form

(13) $$\Delta u=-\nabla (\log\sigma )\cdot \nabla u,x\in {\mathbb{R}}^{2}\backslash \Gamma ,$$

and, because of the assumption that *σ* = 1 on *Γ*, we can formally solve problem (13), (3), by using the fundamental solution

(14) $$\Phi (x,x^{\prime })=\frac{1}{2\pi }\log\frac{1}{\left|x-x^{\prime }\right|}$$

of Laplace's equation (cf., e.g., Kress [6]). This yields

(15) $$\begin{array}{cc} u(x) & =\int_{{ℛ}^{2}\backslash \Gamma }\Phi (x,x^{\prime })\nabla (\log\sigma (x^{\prime }))\cdot \nabla u(x^{\prime })dx^{\prime }\\ & -\int_{\Gamma }\Phi (x,x^{\prime })f(x^{\prime })ds^{\prime }+c, \end{array}$$

where the constant *c* must be chosen so as to satisfy (4). Note that the second term on the right-hand side of (15) is the corresponding potential *u*_0_ associated with the homogeneous reference conductivity. We can therefore rewrite (15) as

(16) $$u(x)=u_{0}(x)+\int_{{\mathbb{R}}^{2}\backslash \Gamma }\Phi (x,x^{\prime })\nabla (\log\sigma (x^{\prime }))\cdot \nabla u(x^{\prime })dx^{\prime }+c.$$

Assuming further that *u* *≈* *u*_0_ we can approximate

(17) $$u(x)-u_{0}(x)\approx \int_{{\mathbb{R}}^{2}\backslash \Gamma }\Phi (x,x^{\prime })\nabla (\log\sigma (x^{\prime }))\cdot \nabla u_{0}(x^{\prime })dx^{\prime }+c,$$

and, since *u*_0_ is a harmonic function and because of our assumption that *σ* = 1 near *Γ* and near infinity, a partial integration of the integral on the right-hand side yields

(18) $$\delta u(x)\approx -\int_{{\mathbb{R}}^{2}\backslash \Gamma }\nabla_{x^{\prime }}\Phi (x,x^{\prime })\cdot \nabla u_{0}(x^{\prime })\log\sigma (x^{\prime })dx^{\prime }+c,$$

where we have set

(19) $$\delta u(x)=u(x)-u_{0}(x).$$

Integrating (18) versus the aforementioned eigenfunctions *f*(^k^) of the reference operator *M*_0_, we get rid of the constant and obtain the system of equations

(20) $$\int_{{\mathbb{R}}^{2}\backslash \Gamma }K_{k}(x^{\prime })\log\sigma (x^{\prime })dx^{\prime }\text{​}=\text{​}\int_{\Gamma }f^{\left(k\right)}(x)\delta u(x)ds,k\in \mathbb{Z}\backslash \{0\},$$

where

(21) $$K_{k}(x)=-\nabla u_{0}^{\left(k\right)}(x)\cdot \nabla u_{0}(x),$$

and $u_{0}^{\left(k\right)}$ is the reference potential corresponding to the input current *f*(^*k*^) (cf. (12)).

We mention that the same linear system is obtained in the first step of Newton's method if the initial conductivity guess is the constant *σ*_0_ = 1, and is to be replaced by *σ*_1_ = 1 + log⁡*σ* which is a good approximation of *σ* if the latter is close to one (cf., e.g., [2] or [7])

**Figure 2:**
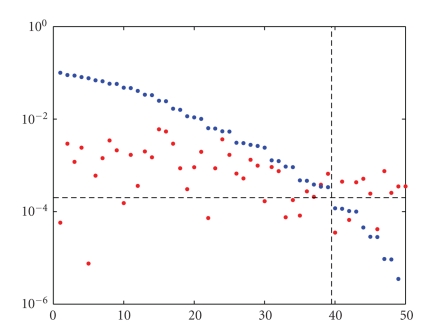
Singular values (blue) and singular components (red) of the right-hand side.

Every boundary current we apply leads to such a system of (20). If we further assume that the conductivity is homogeneous outside of *Γ*, that is, coincides with the background conductivity, then the left-hand side of (20) simplifies to an integral over the unit disk only. The combined set of all these equations can be inverted to obtain the conductivity *σ*. Note, however, that (20) is an approximate identity only, based on the assumption that *u* *≈* *u*_0_. Also, since the problem is ill-posed, we need to regularize this linear system. We implemented the truncated singular value decomposition for this purpose. Figure 2 shows the singular values of the matrix and the corresponding singular components of the right-hand side of (20) for a particular set of data. As indicated by the dashed lines, we used the gap in the singular values near the crossover point with the singular components for truncation.

**Figure 3:**
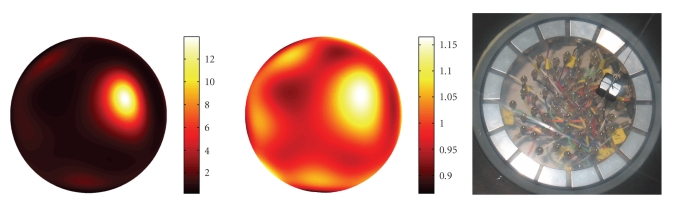
Top: Reconstructions with difference (left) and absolute data (right). Bottom: Phantom used for these two reconstructions.

Figure 2 corresponds to a setup with a metal object of roughly 12 × 13 mm that has been immersed into the container as the phantom to be reconstructed. The phantom and the resulting reconstructions are shown in Figure 3. The left-hand reconstruction has been computed from measured reference potentials *u*_0_ corresponding to a tank with no object immersed. The reconstruction on the right has been computed to simulate a situation where only “absolute” data, that is, the potentials *u*, are available. Here we have approximated *δ* *u* by eliminating the frequency of the injected current from the Fourier spectrum of *u*. Both reconstructions are fairly good, although the one with absolute data is only qualitatively correct.

Note that potentials and currents are only measured on the 16 planar electrodes that are clearly visible in the photo of the phantom.

## 4. THE FACTORIZATION METHOD

Next, we describe the variant of the so-called factorization method which we have implemented for comparison. As a general reference we refer to [8] for more information about this method. In contrast to the previous approach which yields the absolute figures of the conductivity (up to a certain accuracy), this method is not a quantitative one. Instead its purpose is to detect abrupt deviations of the conductivity as compared to a certain reference, namely, the constant background conductivity in our setting. While this approach appears to be quite appropriate for medical applications, it requires difference data and has not yet been generalized to the use of absolute data.

To be precise, we now assume that the true conductivity

(22) $$\sigma (x)=\{\begin{array}{cc} \kappa , & x\in D\subset {\mathbb{R}}^{2},\\ 1, & x\in {\mathbb{R}}^{2}\backslash D, \end{array}$$

is different from the background in some subset *D* ⊂ ℝ ^2^ only. Here, *κ* ≠ 1 is a constant, and *D* is assumed to be strictly on one side of *Γ*, that is, either within the unit disk or completely outside the unit disk. However, *D* need not be connected, but can be the union of finitely many simply connected domains (anomalies, tumors, etc.).

The basic ingredient for the design and analysis of the factorization method is the representation of the relative data

(23) $$M-M_{0}=LFL'$$

as a product of three bounded operators, out of which the first and the last are dual to each other. Recall that *M*_0_ is the measurement operator from (5) associated with the reference conductivity equal to one. The operator *L*, which is the most important for us, is defined via the exterior Neumann problem

(24) $$\Delta w=0\text{ in }{\mathbb{R}}^{2}\backslash \bar{D},\frac{\partial w}{\partial \nu }=\varphi \text{ on }\partial D,$$

with the same constraints

(25) $$\int_{\Gamma }wds=0,|w(x)|=O(1)\text{ as }|x|\to \infty$$

on *w* as in (4); given the solution *w* of (24), (25), the operator *L* now maps

(26) $$L:\varphi \mapsto w{|}_{\Gamma }.$$

Because of our assumption that *D* lies strictly on one side of *Γ*, it can be shown that *L* is injective and its range is dense in ${ℒ}_{⋄}^{2}(\Gamma )$. A more careful analysis which is outside the scope of this paper exhibits the fundamental range identity

(27) $$ℛ(|M-M_{0}{|}^{1/2})=ℛ(L),$$

which is crucial for the success of the factorization method. We refer to [9], or Gebauer [10], for details.

To utilize this result we remark that, on the one hand, the left-hand side member of (27) is available to us because of the given measurements. On the other hand, it is quite easy to characterize the elements of *ℛ*(*L*), the right-hand side member of (27). In fact, consider the potential

(28) $$w_{z,d}(x)=\frac{1}{2\pi }\frac{d\cdot (x-z)}{|x-z{|}^{2}},x\in {\mathbb{R}}^{2}\backslash \{z\},$$

of a dipole in *z* ∈ ℝ^2^\ Γ with dipole moment *d* ∈ ℝ ^2^, |*d*| = 1 , and its trace

(29) $$g_{z,d}=w_{z,d}{|}_{\Gamma }\in {ℒ}_{⋄}^{2}(\Gamma )$$

on Γ. Now it is easy to see that *g*_*z*_,_*d*_ belongs to the range of *L*, if its singularity *z* lies within *D*. Unfortunately, the reverse statement is not quite right. Rather, we have the following theorem.

**Theorem 1.**

Assume that *D* lies strictly on one side of the unit circle Γ, and let *D* ^∗^ be the reflection of *D* with respect to Γ, that is,

(30) $$D^{*}=\{x^{*}=x/|x{|}^{2}:x\in D\backslash \{0\}\}.$$

Then, for *z* ∈ ℝ ^2^, *d* ∈ ℝ ^2^2 with |*d*| = 1, and *g*_*z*_,_*d*_ defined as in (29),

(31) $$g_{z,d}\in ℛ(L)\text{ iff}z\in D\cup D^{*}.$$

**Proof**

The key observation for the proof is that for each dipole potential *w*_*z*_,_*d*_ of (28) with *z* ∉ (Γ ∪ {0}) there exists a corresponding potential *w*_z_^*^,*d*′ of a dipole at z* with dipole moment *d*′ such that the traces of *w*_*z*_,_*d*_ and *w*_z_^*^,*d*′ on Γ are the same. Here, *z*∗ = *z*/|*z*|^2^ is the reflection of *z* with respect to Γ, and *d*′ is given by

(32) $$d^{\prime }=\frac{1}{|z{|}^{4}}(|z{|}^{2}d-2(d\cdot z)z).$$

Since we have observed above that *z* ∈ *D* implies that *g*_*z*_,_*d*_ ∈ *ℛ*(*L*), we are now in the position to add that *g*_*z*_,_*d*_∈ ℛ (*L*), also if *z* ∈ *D*∗. This establishes one direction of the proof. To prove the other direction, assume without loss of generality that *D* is the subset of *D* ∪ *D* ^∗^ that lies within Γ. Next, consider some *z* ∈ ℝ^2^ and some dipole moment *d* for which *g* *_z_*,*_d_* belongs to the range of *L*. Because *w* *_z_*,*_d_* has a pole at *x* = *z*, *z* cannot belong to Γ in this case. We thus assume for the moment that *z* lies within the unit disk. The fact that *g*_*z*_,*_d_* belongs to *ℛ*(*L*) means that there are appropriate Neumann data *φ* such that the unique solution *w* of the exterior Neumann problem (24), (25), and the dipole potential *w*_*z*_,_*d*_ of (28) have the same trace on Γ. As a consequence, *w* and *w*_*z*_,*_d_* both solve the exterior Dirichlet problem

(33) $$\begin{array}{c} \Delta v=0\text{ in}|x|>1,v{|}_{\Gamma }=g_{z,d},\\ |v(x)|=O(1)\text{ for}|x|\to \infty , \end{array}$$

which is known to have a unique solution (cf., e.g., [6]). Hence, *w* = *w* *_z_*,_*d*_, and the unique continuation principle for harmonic functions implies that *w* = *w* *_z_*,_*d*_ in the exterior of *D* ∪ {z}. However, this implies that *z* ∈ *D*, for otherwise the singularity of *w* = *w*_*z*_,*_d_* at *z* would prohibit *w*to be harmonic in ${\mathbb{R}}^{2}\backslash \bar{D}$. In the second case, where *z* is outside the unit disk, we can make use of the remark at the beginning of this proof, which states that *g*_*z*_∗,*d*′ also belongs to ℛ(*L*). Since *z*∗ belongs to the unit disk, the previous argument shows that *z* ^*^ ∈ *D*, or equivalently, *z*∈*D* ^∗^ , in this case. This completes the proof.

By virtue of (27) we can now apply this result to check whether some point *z* belongs to *D* ∪ *D*∗ by means of the following test:

(34) $$z\in D\cup D^{*}\text{ iff}g_{z,d}\in ℛ(|M-M_{0}{|}^{1/2}).$$

Note that the particular choice of the dipole moment *d* is irrelevant for this statement.

We remark that in all applications for which the factorization method has been investigated so far (cf., e.g., the examples in [8, 10, 11]) it was always possible to completely characterize the inclusion set *D*. Here, there is no way to distinguish between *D* and *D* ^∗^. Of course, this handicap can be overcome in practice by moving the device and taking an additional set of measurements.

To implement the factorization method, that is, to test (34) numerically, we can proceed in much the same way as described in [8, 12]. According to the so-called Picard criterion, a function *g*_*z*_,_*d*_ belongs to the range of |*A*|^1^/^2^ for some injective compact and selfadjoint operator *A* (here, *A* = *M* − *M*_0_) if and only if the series

(35) $$\sum_{n=1}^{\infty }\frac{{\langle g_{z,d},v_{n}\rangle }_{{ℒ}^{2}(\Gamma )}^{2}}{\left|\lambda_{n}\right|}$$

converges. Here, {*λ* *_n_*} is the sequence of eigenvalues of *A* and *v*_*n*_ are the associated normalized eigenfunctions. Both can be approximated from the given data. Ordering the eigenvalues *λ* *_n_* such that |*λ*_*n*_| is nonincreasing, the denominators as well as the numerators of the fractions in (35) tend to exhibit a geometric decay. This allows us, for each point *z*∈ ℝ^2^ and each dipole moment *d*∈ ℝ^2^ with |*d*| = 1, to fit two scalar parameters *C*_*z*_,_*d*_ and *q*_*z*_,_*d*_ to achieve

(36) $$\frac{{\langle g_{z,d},v_{n}\rangle }_{{ℒ}^{2}(\Gamma )}^{2}}{\left|\lambda_{n}\right|}\approx C_{z,d}q_{z,d}^{n},n=1,2,3,\ldots ,$$

to a reasonable degree of approximation. Therefore, we can base our numerical implementation of (34) on the following test:

(37) $$z\in D\cup D^{*}\text{ iff}q_{z,d}<1.$$

We refer to [8, 12] for further illustrations and justifications of this test.

Now we turn to some numerical results that have been obtained in [9] this way. In all these computations, test points *z* were aligned on a two-dimensional square grid with a grid size of 1 mm. If not mentioned otherwise, the grid consists of 101 × 101 test points, just covering the circle Γ and its interior.

**Figure 4:**
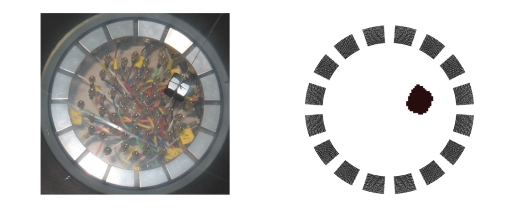
A first reconstruction.

**Figure 5:**
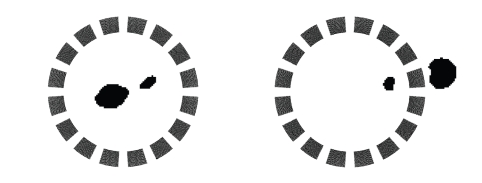
Reconstruction of two objects (left) and a “ghost” (right).

The first example in Figure 4 corresponds to the same measured difference data that we have used for the other algorithm (cf., Figure 3). The black and white plot on the right shows the 16 electrodes together with our reconstruction of the metal object. Figure 5 on the left shows a likewise reconstruction of two objects of sizes 12 × 13 and 20 × 22 mm, respectively. Again, the positions of the reconstructed objects match very well with their true locations, however, their shapes are slightly deteriorated.

Finally, the right-hand side of the same Figure 5 contains the reconstruction of only one object whose position (rather, its projection onto the measurement surface) was *exterior* to the device, that is, the circle Γ. Therefore, the grid is somewhat bigger for this example. The true object is found very well, however, the reconstruction also illustrates Theorem1because a “ghost,” that is, the reflected object *D* ^∗^ in the interior of Γ, has also been detected.

## 5. CONCLUSIONS AND OUTLOOK

In this note, we have presented numerical results for two impedance imaging techniques for a newly built planar EIT device designed primarily for medical applications, where only one side of a patient is accessible for taking measurements.

For our numerical experiments, the sensing head of our device has been attached to a large tank filled with conducting liquid. The arrangement allows a good approximation by an infinite plane. One or two metal objects of different sizes were placed inside the liquid.

We have compared two algorithms for the solution of the inverse problem. The first method is based on a certain kind of linearization, and can be used to reconstruct quantitative information about the conductivity distribution. The numerical results show that this approach is suitable for identifying larger anomalies within the body. For the detection of such anomalies, however, the factorization method is a more appropriate tool, as it is primarily designed for this purpose. In fact, the positions and the sizes of the objects were successfully reconstructed with the factorization method. On the other hand, this method gives no quantitative details about the conductivities. Still, such restricted information is adequate for many medical applications.

In the future, we plan to extend our methods to a fully three-dimensional model of the problem in order to obtain additional information about the depths of the anomalies. The theoretical basis for this has already been laid by Schappel in [13, 14]. Also, the application of our methods to clinical data and the treatment of interrelated side effects like contact impedances at the electrodes is a natural next step on our agenda. We will also exploit the possibility of combining the two reconstruction algorithms in a comprehensive approach.
